# A Novel Segmentation Approach Combining Region- and Edge-Based Information for Ultrasound Images

**DOI:** 10.1155/2017/9157341

**Published:** 2017-04-27

**Authors:** Yaozhong Luo, Longzhong Liu, Qinghua Huang, Xuelong Li

**Affiliations:** ^1^School of Electronic and Information Engineering, South China University of Technology, Guangzhou, China; ^2^Department of Ultrasound, The Cancer Center of Sun Yat-sen University, State Key Laboratory of Oncology in South China, Collaborative Innovation Center for Cancer Medicine, Guangdong, China; ^3^College of Information Engineering, Shenzhen University, Shenzhen 518060, China; ^4^Center for OPTical IMagery Analysis and Learning (OPTIMAL), State Key Laboratory of Transient Optics and Photonics, Xi'an Institute of Optics and Precision Mechanics, Chinese Academy of Sciences, Xi'an, Shaanxi 710119, China

## Abstract

Ultrasound imaging has become one of the most popular medical imaging modalities with numerous diagnostic applications. However, ultrasound (US) image segmentation, which is the essential process for further analysis, is a challenging task due to the poor image quality. In this paper, we propose a new segmentation scheme to combine both region- and edge-based information into the robust graph-based (RGB) segmentation method. The only interaction required is to select two diagonal points to determine a region of interest (ROI) on the original image. The ROI image is smoothed by a bilateral filter and then contrast-enhanced by histogram equalization. Then, the enhanced image is filtered by pyramid mean shift to improve homogeneity. With the optimization of particle swarm optimization (PSO) algorithm, the RGB segmentation method is performed to segment the filtered image. The segmentation results of our method have been compared with the corresponding results obtained by three existing approaches, and four metrics have been used to measure the segmentation performance. The experimental results show that the method achieves the best overall performance and gets the lowest ARE (10.77%), the second highest TPVF (85.34%), and the second lowest FPVF (4.48%).

## 1. Introduction

Ultrasound (US) imaging is one of the most popular medical imaging modalities with numerous diagnostic applications due to the following merits: no radiation, faster imaging, higher sensitivity and accuracy, and lower cost compared to other imaging modalities, such as computed tomography (CT) or magnetic resonance imaging (MRI) [1–6]. However, sonography is operator-dependent, and reading US images requires well-trained and experienced radiologists. To reduce the interobserver variation among different clinicians and help them generate more reliable and accurate diagnostic conclusions, computer-aided diagnosis (CAD) has been proposed [3, 7, 8]. Generally, the CAD system based on the US image involves the following four steps: preprocessing, segmentation, feature extraction and selection, and classification [9, 10]. Among these four procedures, image segmentation which separates the lesion region from the background is the key to the subsequent processing and determines the quality of the final analysis. In the previous clinical practice, the segmentation task is generally performed by manual tracing, which is laborious, time-consuming, and skill- and experience-dependent. Consequently, reliable and automatic segmentation methods are preferred to segment the ROI from the US image, to improve the automation and robustness of the CAD system. However, accurate and automatic US image segmentation remains a challenging task [11–13] due to various US artifacts, including high speckle noise [14], low signal-to-noise ratio, and intensity inhomogeneity [15].

In the last decade, a large number of segmentation methods have been developed for US images, for example, thresholding-based methods [16–18], clustering-based methods [19–23], watershed-based methods [24–27], graph-based methods [28–35], and active contour models [36–42]. Thresholding is one of the frequently used segmentation techniques for the monochrome image. Yap et al. [18] adopted the thresholding segmentation to separate the lesion region from the background before detecting the initial boundary via edge detection. Clustering is a classification technique and has been successfully applied to image segmentation based on similarity between image regions or pixels. Isa et al. [19] used the moving *k*-means clustering to automatically select the seed and proposed a modified seed based region growing algorithm to detect the edge. Shan et al. [20] used a novel neutrosophic clustering approach to detect the lesion boundary. Moon et al. [22] used the fuzzy C-means (FCM) clustering to extract the tumor candidates in their CAD system. The watershed transformation which is frequently used in the segmentation of grey scale images considers the gradient magnitude of an image as a topographic surface. Chen et al. [24] employed the two-pass watershed transformations to generate the cells and proposed a region-based approach called cell-competition algorithm to simultaneously segment multiple objects in a sonogram. L. Zhang and M. Zhang [26] used an extended fuzzy watershed method to segment US images fully automatically. The experiments showed that the proposed method could get good results on blurry US images.

In the last few years, graph-based segmentation has become a research hotspot due to the simple structure and solid theories. In graph-based segmentation, the image is modeled as a weighted, undirected graph. Zhang et al. [28] applied the discriminative graph-cut approach to segmenting tumors after discrimination between tumors and the background via a trained classifier. In 2014, Zhou et al. [29] proposed a novel US image segmentation method based on mean shift and graph cuts (MSGC). It uses mean shift filter to improve the homogeneity and applies graph-cut method, whose energy function combines region- and edge-based information to segment US images. The result showed that the method is rapid and efficient. Huang et al. [30] designed a novel comparison criterion for pairwise subregions which takes local statistics into account to make their method more robust to noises, and hence it was named as robust graph-based (RGB) segmentation method. The experimental results showed that accurate segmentation results can be obtained by this method. However, two significant parameters determining the segmentation result should be set empirically, and for different images they need to be adjusted by repeated tests to obtain good segmentation results. In 2013, Huang et al. [31] proposed an improvement method for RGB by using PSO algorithm to optimize the two significant parameters automatically. The between-class variance, which denotes the difference between the reference region and its adjacent regions, was introduced as the objective function and the method was named as parameter-automatically optimized robust graph-based (PAORGB) segmentation.

The active contour model (ACM), more widely known as snake, is another very popular segmentation method for US images and has been massively used as an edge-based segmentation method. This approach attempts to minimize the energy associated with the initial contour as the sum of the internal and external energies. During the deformation process, the force is calculated from the internal energy and external energy. The internal energy derived from the contour model is used to control the shape and regularity of the contour, and the external energy derived from the image feature is used to extract the contour of the desired object. A 3D snake technique was used by Chang et al. [36] to obtain the tumor contour for the pre- and postoperative malignant tumor excision. Jumaat et al. [37] applied the Balloon Snake to segment the mass in the US image taken from Malaysian population. To overcome the curvature and topology problems in the ACM, level set has been employed to improve the US image segmentation. Sarti et al. [38] used a level set formulation to search the minimal value of ACM, and the segmentation results showed that their model is efficient and flexible. Gao et al. [40] combined an edge stopping term and an improved gradient vector flow snake in the level set framework, to robustly cope with noise and to accurately extract the low contrast and/or concave ultrasonic tumor boundaries. Liu et al. [39] proposed a novel probability density difference-based active contour method for ultrasound image segmentation. In 2010, Li et al. [43] proposed the new level set evolution model Distance Regularized Level Set Evolution (DRLSE) in which it adds a distance regularization term over traditional level set evolution to eliminate the need for reinitialization in evolution process and improve the efficiency. Some researchers combined texture information with other methods for US images segmentation [44–47]. In 2016, Lang et al. [44] used a multiscale texture identifier integrated in a level set framework to capture the spiculated boundary and showed improved segmentation result.

However, most of the above methods are purely region-based or edge-based. For region-based methods, they use homogeneity statistics and low-level image features like intensity, texture, and histogram to assign pixels to objects. Two pixels would be assigned to the same object if they are similar in value and connected to each other in some sense. The problem of applying these approaches to US images is that, without considering any shape information, they would classify pixels within the acoustic shadow as belonging to the tumor, while posterior acoustic shadowing is a common artifact in US images [48, 49]. For edge-based methods (ACM), they are used to handle only the ROI, not the entire image. Although they can obtain the precise contour of the desired object, they are sensitive to noise and heavily rely on the suitable initial contour which is very difficult to generate properly. Also, the deformation procedure is very time-consuming. Therefore, segmentation approaches which integrate region-based techniques and edge-based techniques have been proposed to obtain accurate segmentation results for US images [50–55]. Chang et al. [50] introduced the concepts of 3D stick, 3D morphologic process, and 3D ACM. The 3D stick is used to reduce the speckle noise and enhance the edge information in 3D US images. Then, the 3D morphologic process is used to obtain the initial contour of the tumor for the 3D ACM. Huang and Chen [51, 52] utilized the watershed transform and ACM to overcome the natural properties of US images (i.e., speckle, noise, and tissue-related textures), to segment tumors precisely. In their methods, the watershed transform is performed as the automatic initial contouring procedure for the ACM. Then, the ACM automatically determines the exquisite contour of the tumor. Wang et al. [55] presented a multiscale framework for US image segmentation based on speckle reducing anisotropic diffusion and geodesic active contour. In general, the region-based technique is used to generate the initial contour for the edge-based technique. The experimental results of these approaches indicate that accurate segmentation results can be obtained by combining region-based and edge-based information of the US image.

In this paper, we propose a novel segmentation scheme for US images based on the RGB segmentation method [30] and particle swarm optimization (PSO) algorithm [56, 57]. In this scheme, the PSO is used to optimally set the two significant parameters determining the segmentation result automatically. To combine region-based and edge-based information, we consider the optimization as a multiobjective problem comparing with PAORGB. We use multiobjective optimization method (maximizing the difference between target and background, improving the uniformity within the target region, and considering the edge gradient) to improve the segmentation performance. We take the uniformity of the region and the information of the edge as the objective contents in the process of optimization. First, one rectangle is manually selected to determine the ROI on the original image. However, because of the low contrast and speckle noises of US images, the ROI image is filtered by a bilateral filter and contrast-enhanced by histogram equalization. Next, pyramid mean shift is executed on the enhanced image to improve homogeneity. A novel objective function consisting of three parts corresponding to region-based and edge-based information is designed in the PSO. With the optimization of PSO, the RGB segmentation method is performed to segment the ROI image. Finally, the segmented image is processed by morphological opening and closing to refine the tumor contour.

This paper is organized as follows.  Section 2 introduces the proposed method in detail. Next, the experimental results and comparisons among different methods are presented in Section 3. Finally, we provide some discussion and draw the conclusion in Section 4.

## 2. Methods

**Figure 1:**
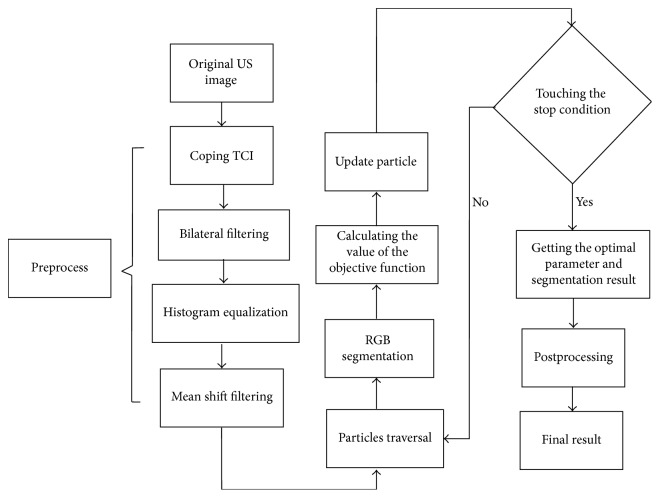
Flowchart of the proposed approach.

In this paper, our method is called multi-objectively optimized robust graph-based (MOORGB) segmentation method, which utilizes PSO algorithm to optimize the two key parameters of RGB segmentation method. In the MOORGB, a multiobjective optimization function which combines region-based and edge-based information is designed in the PSO to optimize the RGB. The flowchart of the proposed approach is shown in Figure 1. In the rest of this section, we introduce each step in the proposed approach in detail.

### 2.1. Preprocessing

#### 2.1.1. Cropping Tumor Centered ROI

**Figure 2:**
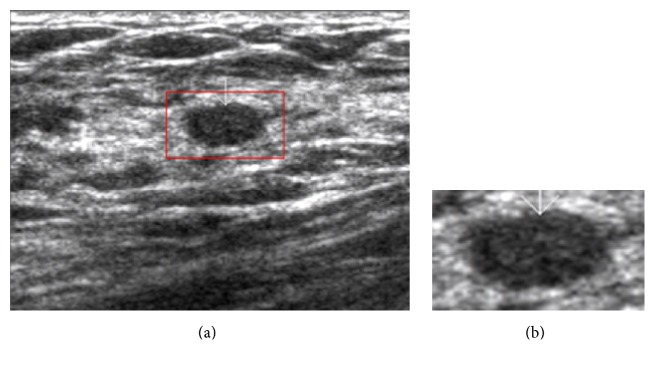
Example of extracting a TCI: (a) the original image and (b) the TCI image.

According to [11] a good segmentation method for clinical US images should have taken advantage of a priori knowledge to improve the segmentation result due to the relatively low quality. In addition, it is hard to describe the segmentation result quantitatively without any a priori knowledge; therefore, it is difficult to design objective function(s) without any a priori knowledge. Therefore, we employ the a priori knowledge used in [31], namely, asking the operator to roughly extract a relatively small rectangular ROI (in which the focus of interest is fully contained and located in the central part) from the US image. In this way, interferences from other unrelated regions can be reduced as much as possible, making the segmentation easier and more efficient. Besides, it gives useful a priori knowledge for design of objective function(s). Such a ROI is called tumor centered image (TCI) in this paper, and Figure 2 shows how a TCI is extracted from a US image.

#### 2.1.2. Bilateral Filtering

Because of diverse interferences (e.g., attenuation, speckle, shadow, and signal dropout) in US images, speckle reduction is necessary to improving the quality of US images. Bilateral filter [58] which has proven to be an efficient and effective method for speckle reduction is adopted in the MOORGB.

#### 2.1.3. Histogram Equalization

To improve the contrast of US images, histogram equalization is conducted to enhance the filtered TCI. Histogram equalization maps one distribution (the given histogram of intensity values in the filtered TCI) to another distribution (a wider and uniform distribution of intensity values). The classical histogram equalization method [59] is used in the MOORGB.

#### 2.1.4. Mean Shift Filtering

**Figure 3:**
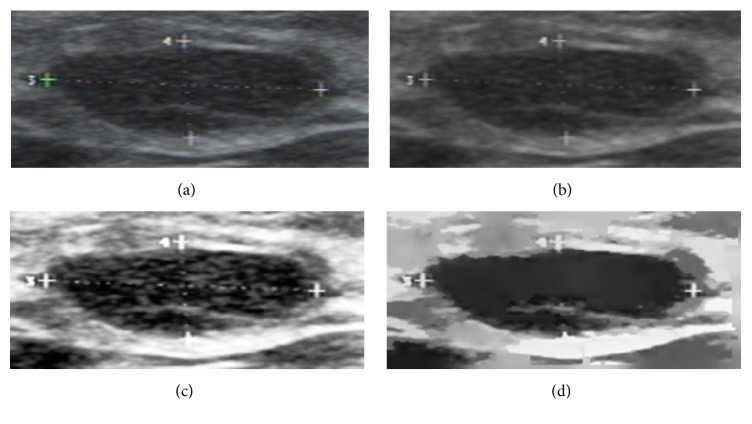
Example of preprocessing, (a) the TCI image, (b) the image after the bilateral filtering, (c) the image after histogram equalization, and (d) the image after mean shift filtering.

After contrast enhancement, we improve the homogeneity by performing mean shift filtering. Mean shift filtering is based on mean shift clustering over grayscale and can well improve the homogeneity of US images and suppress the speckle noise and tissue-related textures [60].  Figure 3 shows the preprocessing results of the image.

### 2.2. RGB Segmentation Method

Given an image which is initially regarded as a graph, the RGB method [30] aims to merge spatially neighboring pixels with similar intensities into a minimal spanning tree (MST), which corresponds to a subgraph (i.e., a subregion in the image). The image is therefore divided into several subregions (i.e., a forest of MSTs). Obviously, the step for merging pixels into a MST is the key, determining the final segmentation results. A novel pairwise region comparison predicate was proposed in the RGB to determine whether or not a boundary between two subgraphs should be eliminated. Given a graph *G* = (*V*, *E*), the resulting predicate *D*(*C*_1_, *C*_2_) which compares intersubgraph differences with within-subgraph differences is formulated as follows [30]:

(1) DC1,C2=false,if  DifC1,C2>MIntC1,C2true,other

(2) DifC1,C2=μC1−μC2

(3) MIntC1,C2=min⁡σC1+τC1,σC2+τC2

(4) τC=kC·1+1α·β,β=μCσC,

where Dif(*C*_1_, *C*_2_) is the difference between two subgraphs, *C*_1_ and *C*_2_ ∈ *V*, MInt(*C*_1_,*C*_2_) represents the smallest internal difference of *C*_1_ and *C*_2_, *μ*(*C*) denotes the average intensity of *C*, *σ*(*C*) is the standard deviation of *C*, and *τ*(*C*) is a threshold function of *C* while *α* and *k* are positive parameters. When *k* increases, *τ* increases as well and the regions merge more easily. On the contrary, when *α* increases, *τ* decreases and hence the regions are merged less easily.

Based on the pairwise region comparison predicate, the general procedures of segmenting an image are as follows.

**Step 1 .**

Construct a graph *G* = (*V*, *E*) for the US image to be segmented. In *G*, each pixel corresponds to a vertex and each edge connects two spatially neighboring vertices. The edge weight is defined by the absolute intensity difference between two adjacent pixels. Initially, each vertex is regarded as a subgraph and all edges constituting the edge set *E* are invalid.

**Step 2 .**

Sort the edges in *E* in nondescending order according to the edge weight, and set *q* = 1.

**Step 3 .**

Pick the *q*th edge in the sorted *E*. If the *q*th edge is an invalid edge (connecting two different subgraphs) and the boundary between these two subgraphs can be eliminated according to the pairwise region comparison predicate as mathematically expressed in (1)–(4); then merge these two subgraphs into a larger subgraph and set this edge valid. Let *q* = *q* + 1.

**Step 4 .**

Repeat Step 3 until all edges in *E* are traversed.

**Figure 4:**
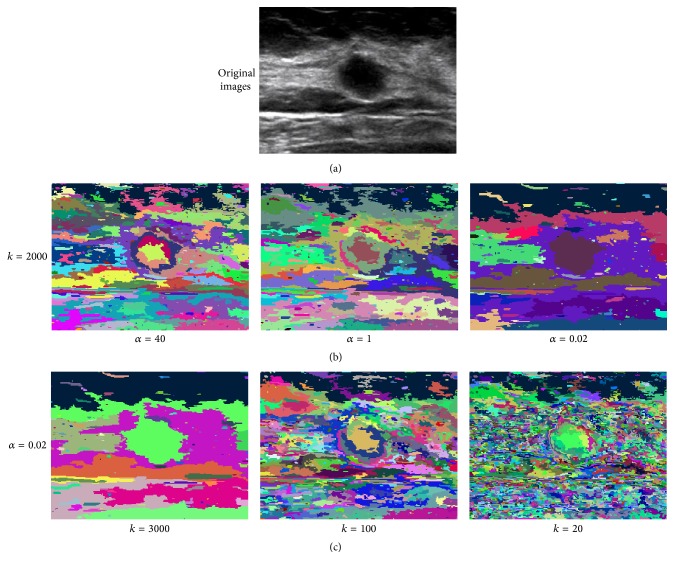
Influence of *α* and *k*: the image of (a) is the original image, the image of (b) shows the segmentation result with different *α*, and (c) shows the segmentation result with different *k*.

When all edges are traversed, a forest including a number of MSTs can be obtained. Each MST corresponds to a subregion in the image. However, the selection of *α* and *k* in (4) can significantly influence RGB's segmentation results [30]. As shown in Figure 4, it can be seen that inappropriate selections of *α* and *k* can lead to under- or oversegmentation. In [30], two significant parameters in RGB segmentation algorithm were empirically selected and usually manually assigned by testing repeatedly to achieve acceptable results. It cannot be fixed for real clinical application because good selections of *α* and *k* may be quite different for different images due to the diversity of US images.

Therefore, the PAORGB was proposed to optimize these two parameters and to achieve a good selection of them automatically for each US image [31]. However, only region-based information and only one optimization goal (maximizing the difference between target and background) have been used. Although the PAORGB can obtain good segmentation results for some US images, its performance is not adequately stable. Therefore, we propose the MOORGB which uses multiobjective optimization method (maximizing the difference between target and background, improving the uniformity within the target region, considering the edge gradient) to improve the segmentation performance. The method makes comprehensive consideration of edge-based and region-based information.

### 2.3. PSO Optimization of Parameters

PSO algorithm is an evolutionary computation technique mimicking the behavior of flying birds and their means of information exchange [56, 57]. In PSO, each particle represents a potential solution, and the particle swarm is initialized with a population of random/uniform individuals in the search space. PSO searches the optimal solution by updating positions of particles in an evolutionary manner.

**Figure 5:**
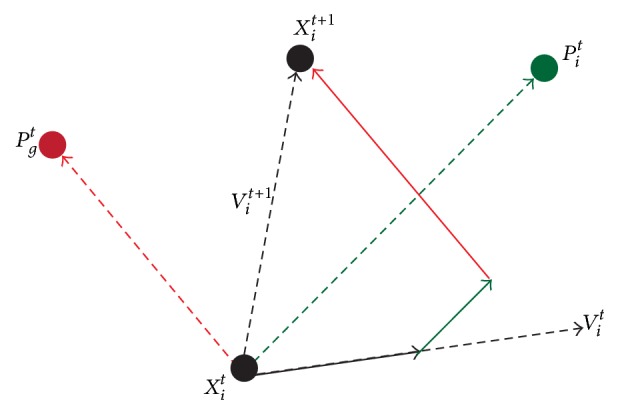
Update of the particle.

Suppose that there are *n*_*p*_ solutions, each of which corresponds to a particle, and the position (i.e., the solution) and velocity of the *i*th particle (*i* = 1,…, *n*_*p*_) are represented by two *m*-dimensional (*m* = 2 in our study) vectors (i.e., *x*_*i*_ = (*x*_*i*1_, *x*_*i*2_,…, *x*_*im*_) and *v*_*i*_ = (*v*_*i*1_, *v*_*i*2_,…, *v*_*im*_), resp.). Position *x* is a vector and in our method, *x* = (*k*, *α*). Velocity *v* means the varied distance of the position at every iteration. *c*_1_, *r*_1_, *c*_2_,  *r*_2_ and *w* are scalars. According to specific issues, one or more objective functions are used to evaluate fitness of each particle, and then the comparison criterion is employed to obtain superior particles. Assume that *p*_*i*_ = (*p*_*i*1_, *p*_*i*2_,…, *p*_*im*_) is the best position visited until the moment of the *i*th particle during the update process, and the global best position of the whole particle swarm obtained so far is indicated as *p*_*g*_ = (*p*_*g*1_, *p*_*g*2_,…, *p*_*gm*_). At each generation, each particle updates its velocity and position according to the following equations after *p*_*i*_ and *p*_*g*_ are acquired through fitness evaluation and the comparison criterion [56]:

(5) vit+1=wvit+c1r1pit−xit+c2r2pgt−xit

(6) xit+1=xit+vit+1

(7) wt=wmax−wmax−wminTmax∗t,

where *t* is the generation number, *T*_max_ is the maximum iteration, *w*^*t*^ is the value of the *t*th iteration, *w* is the inertia weight, *c*_1_ and *c*_2_ are positive parameters known as acceleration coefficients, determining the relative influence of cognition and social components, and *r*_1_ and *r*_2_ are independently uniformly distributed random variables within the range of (0, 1). The value of *w* describes the influence of historical velocity. The method with higher *w* will have stronger global search ability and the method with smaller *w* will has stronger local search ability. At the beginning of the optimization process, we initially set *w* to a large value in order to make better global exploration and gradually decrease it to find optimal or approximately optimal solutions and thus reduce the number of the iterations. Hence we let *w* decrease linearly from 1 towards 0.2, as shown in (7). We set *w*_max_ = 1, *w*_min_ = 0.2, and *T*_max_ = 200. In (5), *wv*_*i*_^*t*^ represents the influence of the previous velocity on the current one, and *c*_1_*r*_1_(*p*_*i*_^*t*^ − *x*_*i*_^*t*^) represents the personal experience while *c*_2_*r*_2_(*p*_*g*_^*t*^ − *x*_*i*_^*t*^) represents the collaborative effect of particles, which pulls particles to the global best solution the whole particle swarm has found so far. As suggested in [41], we set *c*_1_ = 0.5 and *c*_2_ = 0.5 and make personal experience and collaborative effect of particles play the same important role in optimization as shown in Figure 5.

To conclude, at each generation, the velocity and position of each particle are updated according to (5), and its position is updated by (6). At each time, any better position is stored for the next generation. Then, each particle adjusts its position based on its own “flying” experience and the experience of its companions, which means that if one particle arrives at a new promising position, all other particles then move closer to it. This process is repeated until a satisfactory solution is found or a predefined number of iterative generations is met.

The general procedure is summarized as follows.

**Step 1 .**

Properly set the size of particle swarm and randomly/uniformly initialize them according to the search space. In this study, the size of particle swarm is *n*_*p*_ = 200, and the particles are uniformly initialized. According to the work in [30], *k* varies from 100 to 4000 and*α*varies from 0.001 to 4.000, which form the search space.

**Step 2 .**

Traverse all particles: in each traversal (i.e., at each generation), each particle is evaluated through the objective function, and *p*_*i*_ and *p*_*g*_ are acquired according to the comparison criterion.

**Step 3 .**

Update the velocity and position of each particle according to (5) and (6). As suggested in [57], we set *c*_1_ = 0.5 and *c*_2_ = 0.5, and let *w* decrease linearly from 1 towards 0.2.

**Step 4 .**

Repeat Steps 2 and 3 until all particles converge to the predefined extent or the iterative number arrives at the predefined maximum. The predefined extent in this study is that *p*_*g*_ does not change for four iterations, and the maximum iteration is set to *N* = 200 empirically.

### 2.4. The Proposed Objective Function in the PSO

**Figure 6:**
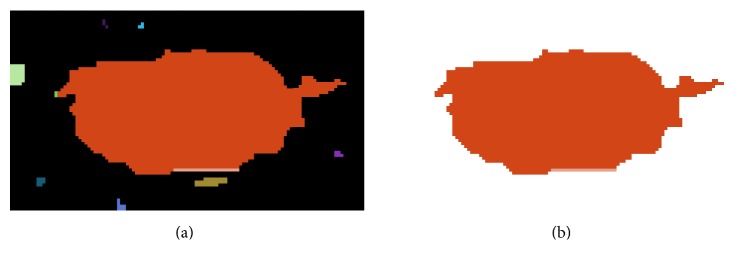
Example of reference region: (a) a segmented image by the RGB and (b) the reference region of (a).

At each time, we use RGB to segment the TCI according to the information (i.e., *α* and *k*) of one particle. According to the a priori knowledge that the focus of interest is located in the central part of TCI, the central subregion with the central pixel of TCI is the possible tumor region. This central subregion is defined as the reference region, which varies with the setting of *α* and *k*, and the reference region is the expected tumor region when *α* and *k* are optimally set.  Figure 6 gives an example of reference region (the original image is shown in Figure 2).

In MOORGB, a novel objective function consisting of three parts corresponding to region-based and edge-based information is adopted. Based on the above a priori knowledge, these three parts, that is, between-class variance, within-class variance, and average gradient, are defined as follows. Compared with PAORGB, we add two objective functions, within-class variance and average gradient. It is not enough to optimize parameters just relying on edge information or region information for segmentation. We take the uniformity of the region and the information of the edge as the objective contents in the optimization process.

#### 2.4.1. Between-Class Variance

Inspired by the idea of Otsu's method [61] which utilizes the difference between subregions to quantitatively describe the segmentation result to select an optimal threshold, the between-class variance (*V*_*B*_) is defined as follows:

(8) $$\begin{array}{c} V_{B}=\overset{k}{\underset{i=1}{\sum }}{P\left(C_{i}\right)\left(\mu \left(C_{i}\right)-\mu \left(C_{Ref}\right)\right)}^{2}, \end{array}$$

where *V*_*B*_ denotes the sum of difference of mean intensity between subregion *C* and the reference region, *k* denotes the number of subregions adjacent to the reference region, and *μ*(*C*) denotes the mean intensity of subregion *C* while *P*(*C*_*i*_) denotes the proportion of the *i*th subregion in the whole TCI and is expressed as

(9) $$\begin{array}{c} P\left(\;C_{i}\;\right)=\frac{\left|C_{i}\right|}{\left|TCI\right|}, \end{array}$$

where |*C*_*i*_| is the number of pixels in the *i*th subregion and |TCI| is the number of pixels in the whole TCI.

From the definition, *V*_*B*_ denotes the difference between the reference region and its adjacent regions. Since the reference region corresponds to the interested tumor region in the US image, it is easy to understand that maximizing *V*_*B*_ can well overcome oversegmentation. By the way, this is the only part adopted in PAORGB [31].

#### 2.4.2. Within-Class Variance

The aim of image segmentation is to segment a region with uniformity, which is always the target object, out of the background [62]. Therefore, considering the uniformity within the target region, we come up with another part called within-class variance (*V*_*W*_) defined as follows:

(10) VW=arctan⁡1/CRef∑i=1CRefIi−μCRef2PCRef,PCRef=CRefTCI,

where |*C*_Ref_| is the number of pixels in the reference region and *I*_*i*_ denotes the intensity of the *i*th pixel while *μ*(*C*_Ref_) denotes the mean intensity of the reference region, and |TCI| is the number of pixels in the whole TCI. Since the minimizing of pure within-class variance (1/|*C*_Ref_|)∑_*i*=1_^|*C*_Ref_|^(*I*_*i*_ − *μ*(*C*_Ref_))^2^ will lead to oversegmentation, we add *P*(*C*_Ref_) to suppress it. Since the value range of (1/|*C*_Ref_|)∑_*i*=1_^*C*_Ref_^(*I*_*i*_ − *μ*(*C*_Ref_))^2^ is much larger than the value range of *P*(*C*_Ref_), we use arctan operation to make them comparable. From the definition, *V*_*W*_ denotes the difference within the reference region, and the undersegmentation problem can be well overcome by minimizing *V*_*W*_.

#### 2.4.3. Average Gradient

As mentioned above, the purpose of segmenting US images is to support the latter analysis and classification in the CAD system, and a wealth of useful and significant information for classification is contained in the contour of the focus. Accordingly, to achieve the objective of acquiring better tumor contours, another part called average gradient (*G*_*A*_) is employed in our objective function. With the inspiration of the definition of energy in ACM, *G*_*A*_ is defined as follows:

(11) $$\begin{array}{c} G_{A}=\frac{1}{m}\overset{m}{\underset{i=1}{\sum }}\left|\;G_{i}\;\right|, \end{array}$$

where *m* is the number of pixels included in the edge of the reference region and *G*_*i*_ denotes the gradient (calculated by the Sobel operator) of the *i*th pixel. Sobel operator is an edge detection operator based on 2D spatial gradient measurement. It can smooth the noise of the image and provide more accurate edge direction information than Prewitt and Roberts operators [59]. *G*_*A*_ denotes the average energy of the edge of the reference region.

Maximizing average gradient *G*_*A*_ obtains more accurate contour and avoids oversegmentation. If there were oversegmentation, the reference region would be included within the real target area; real target area would be a relatively homogeneous region within which every partitioned smaller region would have smaller *G*_*A*_. Consequently, to increase *G*_*A*_ would force the contour of the reference region to move towards that of the real target area. Very often, the edges of the targets in the US image are not sufficiently clear and sharp such that we cannot use only *G*_*A*_ in the objective function. We take average gradient into account as one of the three objective functions in optimization process to improve the segmentation result. In ACM, the initial edge is forced to approach the real edge through maximizing the energy. Similar to ACM, maximizing* G*_*A*_ can force the contour of the reference region to approach the real contour of the tumor.

#### 2.4.4. The Final Objective Function

Based on the above three parts, the objective function is defined as follows:

(12) FO=a∗VBfB−b∗VWfW+c∗GAfA

(13) fB=1np∑i=1npVBi

(14) fW=1np∑i=1npVWi

(15) fA=1np∑i=1npGAi

(16) FO=0.3∗VBfB−0.3∗VWfW+0.4∗GAfA,

where *a*, *b*, *c* are the weights of different objective parts (*a* = 0.3, *b* = 0.3, *c* = 0.4 in our experiment; they can be adjusted as needed). The final objective function in the experiment is defined as (16). *V*_*B*_, *V*_*W*_, and *G*_*A*_ are between-class variance, within-class variance, and average gradient, respectively. *f*_*B*_, *f*_*W*_, and *f*_*A*_ are normalized factors while *n*_*p*_ = 200 is the size of particle swarm. Because the value ranges of *V*_*B*_, *V*_*W*_, and *G*_*A*_ are quite different, they should be normalized to be comparable. For each US image, *f*_*B*_, *f*_*W*_, and *f*_*A*_ are calculated once after the uniform initialization of particle swarm but before the first iteration. We try to maximize *F*_*O*_ by the PSO.

### 2.5. Postprocessing

After the TCI is segmented by the RGB with the optimal *α* and *k* obtained by the PSO, we turn it into a binary image containing the object (tumor) and the background (tissue). Next, morphological opening and closing are conducted to refine the tumor contour, with opening to reduce the spicules and closing to fill the holes. A 5 × 5 elliptical kernel is used for both opening and closing.

### 2.6. The Proposed MOORGB Segmentation Method

Assuming that the position and velocity of the *i*th particle in our case are expressed as *x*_*i*_ = (*k*_*i*_, *α*_*i*_) and *v*_*i*_ = (*v*_*ki*_, *v*_*αi*_), respectively, the general procedure of MOORGB is summarized as follows.

**Step 1 .**

Manually delineate TCI from the original US image.

**Step 2 .**

Use the bilateral filter to do the speckle reduction for TCI.

**Step 3 .**

Enhance the filtered TCI by histogram equalization to improve the contrast.

**Step 4 .**

Improve the homogeneity by performing pyramid mean shift filtering.

**Step 5 .**

Uniformly initialize the particle swarm within the search space, and let the iteration count *q* = 0 and so on.

**Step 6 .**

Let *q* = *q* + 1; traverse all *n*_*p*_ particles: in the *q*th traversal, RGB is performed with the position (i.e., *x*_*i*_ = (*k*_*i*_, *α*_*i*_)) of each particle; then evaluate the segmentation result with the objective function *F*_*O*_ and obtain *p*_*i*_ and *p*_*g*_ by comparing values of *F*_*O*_ for updating each particle (including position and velocity) for next iteration.

**Step 7 .**

Iteratively repeat Step 6 until convergence (i.e., *p*_*g*_ remains stable for 4 generations) or *q* = *N* (*N* = 200 in this paper).

**Step 8 .**

After finishing the iteration, the position of the globally best particle (i.e., *p*_*g*_) is, namely, the optimal setting of *α* and *k*; then get the final segmentation result by performing RGB with the optimal setting.

**Step 9 .**

Turn the segmentation result into a binary image; then get the final tumor contour by conducting morphological opening and closing.

### 2.7. Experimental Methods

We developed the proposed method with the C++ language using OpenCV 2.4.3 and VisualStudio 2010 and run it on a computer with 3.40 GHz CPU and 12.0 GB RAM. To validate our method, experiments have been conducted. Our work is approved by Human Subject Ethics Committee of South China University of Technology. In the dataset, 100 clinical breast US images and 18 clinical musculoskeletal US images with the subjects' consent forms were provided by the Cancer Center of Sun Yat-sen University and were taken from an HDI 5000 SonoCT System (Philips Medical Systems) with an L12-5 50 mm Broadband Linear Array at the imaging frequency of 7.1 MHz. The “true” tumor regions of these US images were manually delineated by an experienced radiologist who has worked on US imaging and diagnosis for more than ten years. The contour delineated by only one doctor is not absolutely accurate because different doctors may give different “real contours,” which is indeed a problem in the research. Nevertheless, the rich diagnosis experience of the doctor has fitted the edge of every tumor as accurately as possible. This dataset consists of 50 breast US images with benign tumors, 50 breast US images with malignant tumors, and 18 musculoskeletal US images with cysts (including 10 ganglion cysts, 4 keratinizing cysts, and 4 popliteal cysts).

To demonstrate the advantages of the proposed method, besides PAORGB, we also compared the method with the other two well-known segmentation methods (i.e., DRLSE [43] and MSGC [29]). DRLSE method, an advanced level set evolution approach in recent years, is applied to an edge-based active contour model for image segmentation. It is an edge-based segmentation method that needs to set initial contour manually. The initial contour profoundly affects the final segmentation result. MSGC is a novel graph-cut method whose energy function combines region- and edge-based information to segment US images. It also needs to crop tumor centered ROI. Among the three comparative methods, DRLSE is an edge-based method, PAORGB is a region-based method, and MSGC is a compound method. To make a comparison of computational efficiency, the methods PAORGB and MOORGB were programmed in the same software system. As such, the four methods were run with the same hardware configuration. The ROI is all the same for the four segmentation methods.

**Figure 7:**
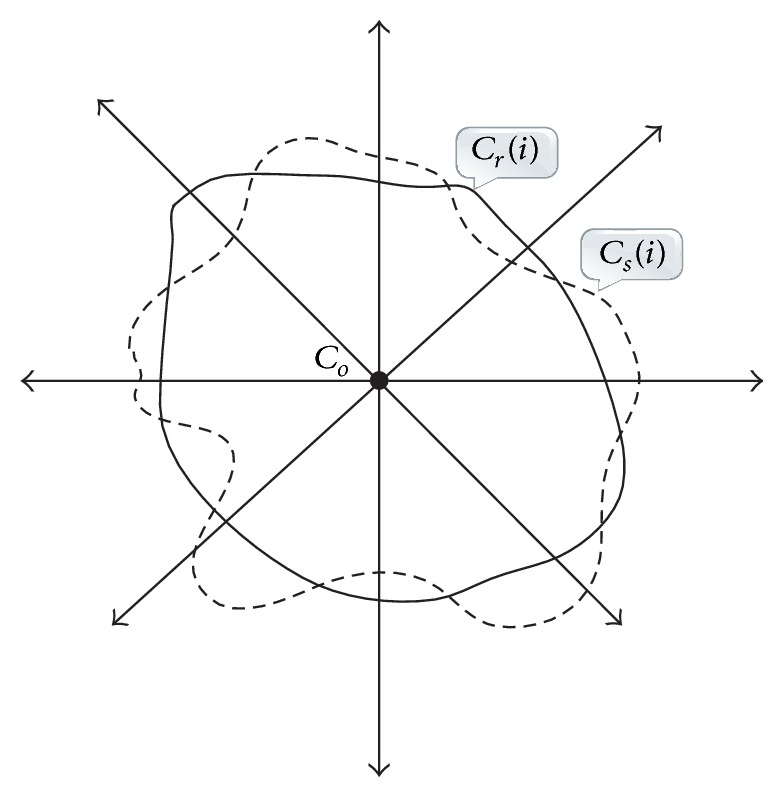
An illustration of computation principle for ARE.

To quantitatively measure the experiment results, four criteria (i.e., averaged radial error (ARE), true positive volume fraction (TPVF), false positive volume fraction (FPVF), and false negative volume fraction (FNVF)) were adopted in this study. The ARE is used for the evaluation of segmentation performance by measuring the average radial error of a segmented contour with respect to the real contour which is delineated by an expert radiologist. As shown in Figure 7, it is defined as

(17) $$\begin{array}{c} \text{ARE}\left(\;n\;\right)=\frac{1}{n}\overset{n-1}{\underset{i=0}{\sum }}\frac{\left|C_{s}\left(i\right)-C_{r}\left(i\right)\right|}{\left|C_{r}\left(i\right)-C_{o}\right|}\times 100\%, \end{array}$$

where *n* is the number of radial rays and set to 180 in our experiments while *C*_*o*_ represents the center of the “true” tumor region which is delineated by the radiologist and *C*_*s*_(*i*) denotes the location where the contour of the segmented tumor region crosses the *i*th ray, while *C*_*r*_(*i*) is the location where the contour of the “true” tumor region crosses the *i*th ray.

**Figure 8:**
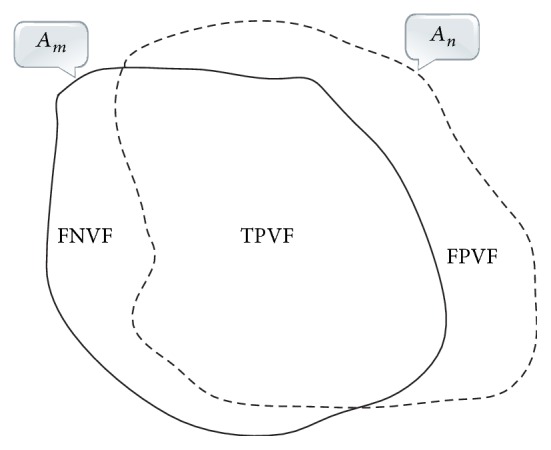
The areas corresponding to TPVF, FPVF, and FNVF, respectively. *A*_*m*_ indicates the “true” contour delineated by the radiologist and *A*_*n*_ denotes the contour obtained by the segmentation algorithm.

In addition, TPVF, FPVF, and FNVF were also used in the evaluation of the performance of segmentation methods. TPVF means true positive volume fraction, indicating the total fraction of tissue in the “true” tumor region with which the segmented region overlaps. FPVF means false positive volume fraction, denoting the amount of tissue falsely identified by the segmentation method as a fraction of the total amount of tissue in the “true” tumor region. FNVF means false negative volume fraction, denoting the fraction of tissue defined in the “true” tumor region that is missed by the segmentation method. In our study, the “true” tumor region is delineated by the radiologist.  Figure 8 shows the areas corresponding to TPVF, FPVF, and FNVF. Accordingly, smaller ARE, FPVF, and FNVF and larger TPVF indicate better segmentation performance. TPVF, FPVF, and FNVF are defined by

(18) TPVF=Am∩AnAmFPVF=An−Am∩AnAmFNVF=Am−Am∩AnAm,

where *A*_*m*_ is the area of the “true” tumor region delineated by the radiologist and *A*_*n*_ is the area of the tumor region obtained by the segmentation algorithm.

## 3. Experimental Results and Discussion

### 3.1. Qualitative Analysis

**Figure 9:**
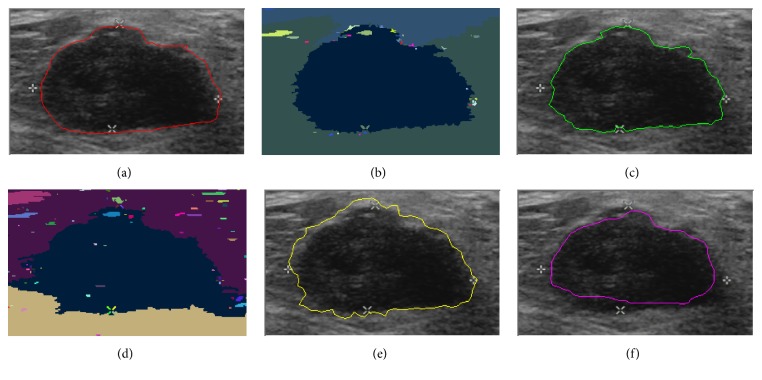
Segmentation results for the first benign breast tumor. (a) A breast US image with a benign tumor (the contour is delineated by the radiologist). (b) The result of MOORGB. (c) The final result of MOORGB. (d) The result of PAORGB. (e) The result of DRLSE. (f) The result of MSGC.

**Figure 10:**
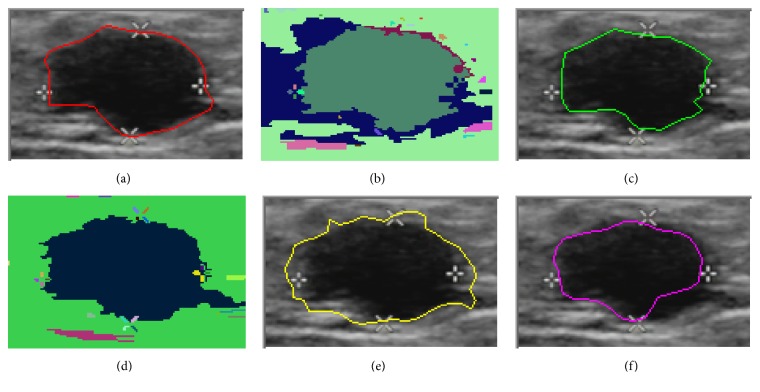
Segmentation results for the second benign breast tumor. (a) A breast US image with a benign tumor (the contour is delineated by the radiologist). (b) The result of MOORGB. (c) The final result of MOORGB. (d) The result of PAORGB. (e) The result of DRLSE. (f) The result of MSGC.

**Figure 11:**
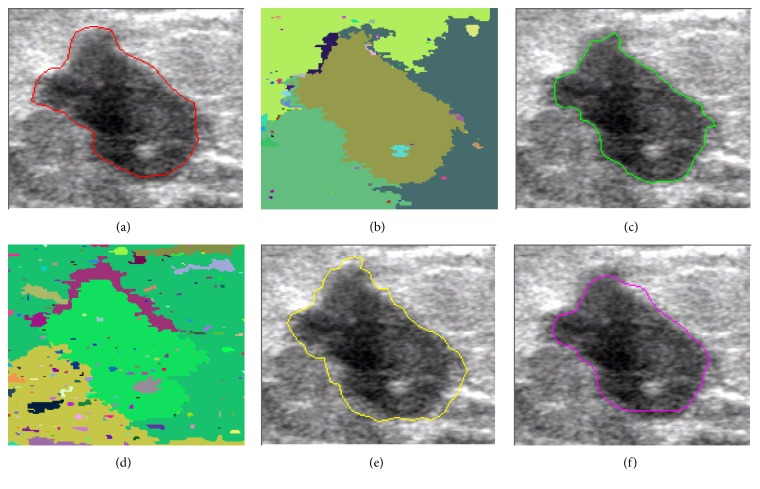
Segmentation results for the first malignant breast tumor. (a) A breast US image with a malignant tumor (the contour is delineated by the radiologist). (b) The result of MOORGB. (c) The final result of MOORGB. (d) The result of PAORGB. (e) The result of DRLSE. (f) The result of MSGC.

**Figure 12:**
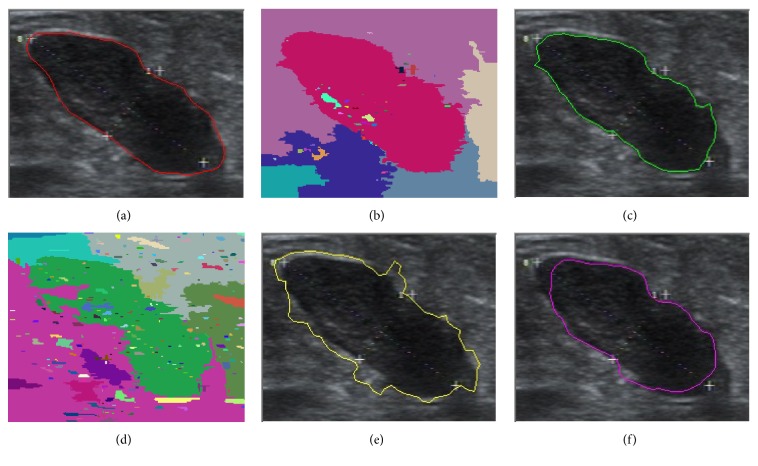
Segmentation results for the second malignant breast tumor. (a) A breast US image with a malignant tumor (the contour is delineated by the radiologist). (b) The result of MOORGB. (c) The final result of MOORGB. (d) The result of PAORGB. (e) The result of DRLSE. (f) The result of MSGC.

**Figure 13:**
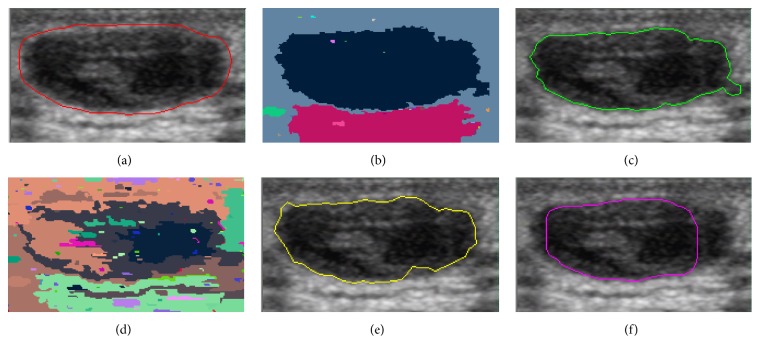
Segmentation results for the keratinizing cyst. (a) A musculoskeletal US image with a cyst (the contour is delineated by the radiologist). (b) The result of MOORGB. (c) The final result of MOORGB. (d) The result of PAORGB. (e) The result of DRLSE. (f) The result of MSGC.

In this paper, we present the segmentation results for five tumors. Five US images with the segmentation results are shown in Figures 9–13. The quantitative segmentation results on US images are shown in Tables 1, 2, 3, and 4. Figures 9(a), 10(a), 11(a), 12(a), and 13(a) show original B-mode US images for two benign tumors, two malignant tumors, and one musculoskeletal cyst, respectively. After preprocessing the original images, the segmentation results using the MOORGB are illustrated in Figures 9(b), 10(b), 11(b), 12(b), and 13(b), those using the PAORGB in Figures 9(d), 10(d), 11(d), 12(d), and 13(d), those using the DRLSE in Figures 9(e), 10(e), 11(e), 12(e), and 13(e), and those using the MSGC in Figures 9(f), 10(f), 11(f), 12(f), and 13(f).

In Figures 9–13, we can see that our method achieved the best segmentation results compared with the other three methods, and the contour generated by our method is quite close to the real contour delineated by the radiologist. Undersegmentation happens in Figures 9(d) and 10(d), but not in Figures 9(b) and 10(b); and oversegmentation happens in Figures 12(d) and 13(d), but not in Figures 12(b) and 13(b). Comparing with the PAORGB, the MOORGB improves the segmentation results obviously, avoiding the undersegmentation and oversegmentation more effectively. Regional uniformity has been significantly improved, and the edge has been smoother. The reason is that the within-class variance and average gradient are introduced into the objective function of MOORGB by combining region- and edge-based information. The segmentation results of the MSGC are better than those of the PAORGB and DRLSE, since MSGC is also a compound method (region energy and boundary energy are both included in its energy function) and many preprocessing techniques are adopted. As shown in Figures 9(e), 10(e), 11(e), 12(e), and 13(e), the DRLSE can only roughly detect the tumor contour, and the detected contours are irregular. The reason is that it depends on edge-based information and is sensitive to speckle noise and sharp edge; hence it captures sharp edge easily and leads to boundary leakage and undersegmentation.

### 3.2. Quantitative Analysis

Table 4 shows the quantitative comparisons of different segmentation approaches on the whole dataset. Similarly, we show the quantitative segmentation results of the benign tumors, malignant tumors, and cysts in Tables 1, 2, and 3, respectively.

Comparing Tables 1 and 3 with Table 2, it is shown that all four segmentation methods perform better on benign tumors and musculoskeletal cysts than on malignant tumors on the whole, indicating that the boundaries of benign tumors and musculoskeletal cysts are more significant than those of malignant tumors. The shape of the benign tumor is more regular and similar to circle or ellipse. The shape of the malignant tumor is irregular and usually lobulated with burrs in the contour. The segmentation result of the malignant tumor is worse than benign tumors because the contour of malignant tumor is less regular and less homogenous than that of benign tumor.

From Table 4, it is seen that our method achieved the lowest ARE (10.77%). Due to the undersegmentation, the DRLSE got the highest TPVF (94.07%) and FPVF (17.97%), indicating the high ratio of false segmentation. The MSGC got the lowest FPVF (2.9%), which indicates the low ratio of false segmentation, and is the fastest method (0.123 s) among the four methods. However it got the lowest TPVF (75.61%), showing oversegmentation in a way. Comparing with the original PAORGB (as shown in Table 4), our method improves the segmentation result obviously, achieving higher TPVF and lower ARE, FPVF, and FNVF. Although MOORGB could not achieve the best performance in all evaluation indices, it got the best overall performance. Comparing with DRLSE, our method was 6.45% lower than it in TPVF but 8.75% better than it in FPVF, obtaining better overall performance. Comparing with MSGC, our method was 1.58% higher than it in FPVF but 9.93% better than it in TPVF, obtaining better overall performance and avoiding oversegmentation in a way. As shown in Table 4, our method is faster than the PAORGB. It is because the convergence condition in our method is that “*p*_*g*_ remains stable for 4 generations,” rather than that “the updating of *k* is below 1 and that of *α* is below 0.00001 for all the particles in an experiment” in the PAORGB [31].

### 3.3. The Influence of the Weight

**Figure 14:**
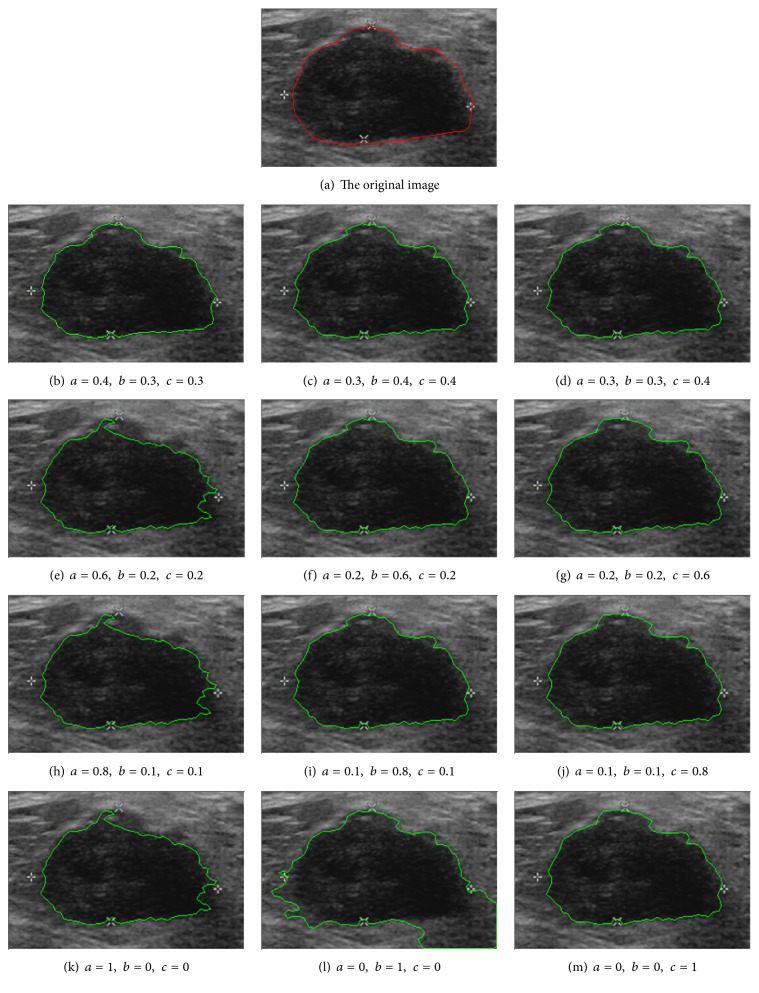
The segmentation results with different weights (*a*, *b*, *c*).

Our method synthesizes three optimization objective functions (between-class variance *V*_*B*_, within-class variance *V*_*W*_, and average gradient *G*_*A*_). Thus the weight values of three objective parts (i.e., *a*, *b*, and *c*) are introduced.  Figure 14 shows the comparison of experimental results with different weight values. From Figures 14(a), 14(b), and 14(c) and Table 5, we can see that when the weight values of the three objective functions are almost the same, three optimization objectives play nearly equal roles in the optimization process, making the algorithm not only region-based but also edge-based. When one of the three weight values is overlarge, it would not be able to reflect the three optimization results evenly, hence leading to oversegmentation or undersegmentation. As shown in Figures 14(k), 14(l), and 14(m), if one of the three weight values equals one, the proposed method degenerated into a single objective optimization algorithm, and the optimization goal is only one role, which cannot avoid oversegmentation and undersegmentation effectively. Through analyzing the influence of different weight values of objective functions and our repeated experiments, we can set the system parameters as *a* = *b* = 0.3, *c* = 0.4. The final objective function is described as (16) which can make segmentation system work well.

## 4. Conclusions

In this paper, we propose a novel segmentation scheme for US images based on the RGB and PSO methods. In this scheme, the PSO is used to optimally set the two significant parameters in the RGB for determining the segmentation result automatically. To combine region-based and edge-based information, we consider the optimization as a multiobjective problem. First, because of the low contrast and speckle noises of US images, the ROI image is filtered by a bilateral filter and contrast-enhanced by histogram equalization and then pyramid mean shift is executed on the enhanced image to improve homogeneity. A novel objective function consisting of three parts corresponding to region-based and edge-based information is adopted by PSO. The between-class variance denotes the difference between the reference region and its adjacent regions. The within-class variance denotes the difference within the reference region, and the undersegmentation problem can be well overcome by minimizing it. Between-class variance and within-class variance reflect the regional information. The average gradient denotes the average energy of the edge of the reference region and maximizing it can force the contour of the reference region to approach the real contour of the tumor. Average gradient reflects the edge-based information of the image. Three optimization objectives play important roles in the optimization process, making the algorithm achieve the corresponding segmentation effect, not only region-based but also edge-based. With the optimization of PSO, RGB is performed to segment the ROI image. Finally, the segmented image is processed by morphological opening and closing to refine the tumor contour. To validate our method, experiments have been conducted on 118 clinical US images, including breast US images and musculoskeletal US images. The segmentation results of our method have been compared with the corresponding results obtained by three existing approaches, and four metrics have been used to measure the segmentation performance. The experimental results show that our method could successfully segment US images and achieved the best segmentation results compared with the other three methods, MSGC, PAORGB, and DRLSE. The contour generated by our method was closer to the real contour delineated by the radiologist. The MOORGB obtained the lowest ARE and better overall performance in TPVF, FPVF, and FNVF, avoiding the undersegmentation and oversegmentation more effectively.

However, the step to obtain TCI (as shown in Figure 2) requires user's participation which may result in significant influence on the following segmentation. To obtain acceptable segmentation results, the operator should be well experienced in examining US images and identifying suspicious lesions in clinical practices. Moreover, the TCI should be carefully delineated to achieve the full lesion region with partial surrounding tissues, and the interested lesion region must be located in the central part. Consequently, how to automatically extract TCI from the BUS image is one of our future studies. In addition, the computation time is still far away from real-time applications. Accordingly, making efforts to reduce the computation time by adopting parallel processing techniques is also part of our future work. Besides, adopting our segmentation method in real CAD systems to validate the whole performance will be included in our future work.

## Figures and Tables

**Table 1:** Quantitative segmentation results of 50 breast US images with benign tumors.

Methods	ARE (%)	TPVF (%)	FPVF (%)	FNVF (%)
Our method	11.09 ± 12.47	85.60 ± 13.71	4.51 ± 20.18	14.40 ± 13.71
PAORGB [26]	16.47 ± 21.41	81.64 ± 29.94	10.52 ± 29.40	18.36 ± 29.94
DRLSE [46]	11.37 ± 13.04	93.60 ± 16.87	14.42 ± 24.33	6.40 ± 16.87
MSGC [24]	15.76 ± 13.18	75.34 ± 16.25	2.51 ± 14.60	24.66 ± 16.24

**Table 2:** Quantitative segmentation results of 50 breast US images with malignant tumors.

Methods	ARE (%)	TPVF (%)	FPVF (%)	FNVF (%)
Our method	10.41 ± 13.62	84.91 ± 16.39	4.43 ± 19.01	15.09 ± 16.39
PAORGB	19.12 ± 27.63	74.98 ± 27.49	10.16 ± 37.09	25.02 ± 27.49
DRLSE	15.84 ± 15.34	95.31 ± 19.75	24.05 ± 20.68	4.69 ± 19.75
MSGC	15.52 ± 22.66	74.12 ± 15.12	2.93 ± 13.17	25.88 ± 15.12

**Table 3:** Quantitative segmentation results of 18 musculoskeletal US images with cysts.

Methods	ARE (%)	TPVF (%)	FPVF (%)	FNVF (%)
Our method	10.85 ± 17.14	85.61 ± 7.75	4.52 ± 34.22	14.30 ± 7.80
PAORGB	20.90 ± 39.66	82.12 ± 29.33	18.00 ± 35.97	17.80 ± 29.40
DRLSE	8.60 ± 12.06	91.90 ± 21.61	10.90 ± 10.72	8.04 ± 21.66
MSGC	14.43 ± 27.30	80.50 ± 17.33	3.9 ± 43.41	19.35 ± 29.65

**Table 4:** Overall quantitative segmentation results of 118 US images.

Methods	ARE (%)	TPVF (%)	FPVF (%)	FNVF (%)	Averaged computing time (s)
Our method	10.77 ± 17.22	85.34 ± 16.69	4.48 ± 34.26	14.67 ± 16.67	50.54
PAORGB	18.27 ± 37.03	78.89 ± 30.24	10.51 ± 35.74	21.10 ± 30.23	719.78
DRLSE	12.84 ± 16.82	94.07 ± 18.51	17.97 ± 28.03	5.92 ± 23.78	5.93
MSGC	15.46 ± 26.27	75.61 ± 17.74	2.9 ± 42.41	24.37 ± 24.63	**0.123**

**Table 5:** Quantitative segmentation results of 15 US images with different weight values.

Methods	ARE (%)	TPVF (%)	FPVF (%)	FNVF (%)
*a* = 0.4, *b* = 0.3, *c* = 0.3	10.67	85.61	4.52	14.70
*a* = 0.3, *b* = 0.4, *c* = 0.3	10.71	85.60	4.51	14.69
*a* = 0.3, *b* = 0.3, *c* = 0.4	10.69	85.60	4.52	14.69
*a* = 0.6, *b* = 0.2, *c* = 0.2	10.47	85.51	4.49	14.91
*a* = 0.2, *b* = 0.6, *c* = 0.2	10.59	85.52	4.50	14.73
*a* = 0.2, *b* = 0.2, *c* = 0.6	10.74	85.64	4.80	14.75
*a* = 0.8, *b* = 0.2, *c* = 0.2	11.12	84.97	5.44	15.39
*a* = 0.2, *b* = 0.8, *c* = 0.2	11.25	85.03	5.78	15.41
*a* = 0.2, *b* = 0.2, *c* = 0.8	11.23	84.77	5.61	15.28
*a* = 1, *b* = 0, *c* = 0	12.92	83.39	6.78	16.07
*a* = 0, *b* = 1, *c* = 0	69.84	98.86	154.72	0.46
*a* = 0, *b* = 0, *c* = 1	8.97	87.79	10.49	17.93
